# High levels of pesticides found in illicit cannabis inflorescence compared to licensed samples in Canadian study using expanded 327 pesticides multiresidue method

**DOI:** 10.1186/s42238-023-00200-0

**Published:** 2023-08-24

**Authors:** Mathieu Gagnon, Tyler McRitchie, Kim Montsion, Josée Tully, Michel Blais, Neil Snider, David R. Blais

**Affiliations:** Pesticide Laboratory, Regulatory Operations and Enforcement Branch, Health Canada, Ottawa, ON K1A 0C6 Canada

**Keywords:** QuEChERS, Gas chromatography–triple quadrupole mass spectrometry, Liquid chromatography–triple quadrupole mass spectrometry, Cannabis inflorescence, Pesticides, Licensed versus Illicit

## Abstract

**Background:**

As Cannabis was legalised in Canada for recreational use in 2018 with the implementation of the *Cannabis Act*, *Regulations* were put in place to ensure safety and consistency across the cannabis industry. This includes the requirement for licence holders to demonstrate that no unauthorized pesticides are used to treat cannabis or have contaminated it. In this study, we describe an expanded 327 multi-residue pesticide analysis in cannabis inflorescence to confirm if the implementation of the *Cannabis Act* is providing safer licensed products to Canadians in comparison to those of the illicit market.

**Methods:**

An extensive multi-residue method was developed using a modified quick, easy, cheap, effective, rugged, and safe (QuEChERS) sample preparation method using a combination of gas chromatography—triple quadrupole mass spectrometry (GC–MS/MS) and liquid chromatography—triple quadrupole mass spectrometry (LC–MS/MS) for the simultaneous quantification of 327 pesticide active ingredients in cannabis inflorescence.

**Results:**

Application of this method to Canadian licensed inflorescence samples revealed a 6% sample positivity rate with only two pesticide residues detected, myclobutanil, and dichlobenil, at the method’s lowest calibrated level (LCL) of 0.01 μg/g. Canadian illicit cannabis inflorescence samples analysed showed a striking contrast with a 92% sample positivity rate covering 23 unique pesticide active ingredients with 3.7 different pesticides identified on average per sample. Chlorpyrifos, imidacloprid, and myclobutanil were measured in illicit samples at concentrations up to three orders of magnitude above the method LCL of 0.01 μg/g.

**Conclusion:**

These results demonstrate the need of an extensive multiresidue method capable of analysing hundreds of pesticides simultaneously, to generate data for future policy and regulatory decision-making, and to enable Canadians to make safe cannabis choices.

**Supplementary Information:**

The online version contains supplementary material available at 10.1186/s42238-023-00200-0.

## Background

In 2018, Canada legalised the recreational usage of cannabis, supplementing cannabis for medical purposes framework, which had been in place since 2001. The coming into force of the *Cannabis Act* (Cannabis Act 2018) and its *Regulations* (Cannabis Regulations 2018) aims at standardizing and enforcing consistency, health, and safety across Canada’s legal cannabis industry. To ensure safe cannabis products to Canadians, Health Canada regulates microbial and chemical contaminants, including pesticides. In addition to the existing analytical testing requirements under the *Cannabis Regulations*, since January 2019, the industry must also follow the Mandatory cannabis testing for pesticide active ingredients requirements (Mandatory cannabis testing for pesticide active ingredients requirements 2019) where Licence holders must demonstrate that none of the 96 unauthorized pesticide active ingredients are used to treat cannabis or have contaminated it.

With over 18% of licensed cannabis products containing unregistered pesticides prior to the 2019 mandatory cannabis testing of 96 pesticide active ingredients, with myclobutanil, bifenazate, boscalid, and fludioxonil pesticides most commonly present (Moulins et al. 2018), and for which myclobutanil is classified moderately hazardous by the World Health Organization (WHO 2019). This study aims to determine if unregistered pesticides are still prevalent in the licensed market. To gain a broader view of pesticide usage during cannabis production, we streamlined, expanded, and validated a single method using a combination of gas chromatography—triple quadrupole mass spectrometry (GC–MS/MS) and liquid chromatography—triple quadrupole mass spectrometry (LC–MS/MS) for the simultaneous quantification of 327 pesticide active ingredients in cannabis inflorescence, that goes well beyond the 96 pesticide active ingredients mandatory testing (Mandatory cannabis testing for pesticide active ingredients requirements 2019). Although the cannabis licensed market has been gaining ground in Canada since legalisation, up to 13% of Canadians still report consuming illicit cannabis almost exclusively (Canadian Cannabis Survey 2021). Illicit cannabis samples were also analysed for pesticides to determine how they compare to the Canadian licensed cannabis market.

## Methods

### Sampling

To reflect as realistically as possible the sources of cannabis inflorescence available to Canadians across the country, 36 licensed samples were purchased in 2021 from the Ontario Cannabis Store (Ontario, Canada) from licence holders located in all five Canadian regions (British Columbia, Prairies, Ontario, Quebec, and Atlantic) (Table 1). The 24 illicit cannabis samples were obtained from seizures by law enforcement officers across the country and submitted to Health Canada for laboratory testing in 2021.

**Table 1:** Geographical distribution of cannabis (*C. sativa*) inflorescence samples obtained across Canada

Region	Licensed samples	Illicit samples
British Columbia	9	3
Prairies	5	1
Ontario	12	5
Québec	6	14
Atlantic	4	1
**Total**	**36**	**24**

### Standards and reagents

Pesticide analytical standards were purchased from Chemservice (West Chester, PA) and Sigma-Aldrich Canada (Oakville, ON). Analytical grade acetone and toluene were purchased from EMD Millipore (Darmstadt, Germany). Analytical grade acetonitrile and Na_2_SO_4_ were purchased from Fisher Scientific (Fairlawn, NJ). Water was obtained from a Milli-Q® Plus Ultra Pure Water system (Millipore Corp., Burlington, MA). Sepra™C18-E was obtained from Phenomenex (Torrance, CA). Supelclean™ ENVI™-Carb SPE Tubes were obtained from Supelco (Bellefonte, PA). Sep-Pak® Classic NH2 Cartridges were obtained from Waters Corp. (Milford, MA).

### Apparatus

For sample preparation, a laboratory blender 51BL30 (Stamford, Connecticut), a high-speed shaker (Spex Sample Prep Geno-Grinder; Fisher Scientific, Fairlawn, NJ), a centrifuge (Allegra X15R 208v; Beckman Coulter Inc., Brea, CA), a solvent evaporator (Xcelvap; Horizon Technologies, Salem, NH), and a rotary evaporator (Rotavpor R-114, BÜCHI Labortechnik AG, Flawil, Switzerland) were used. Sample analysis was carried out on a GC–MS/MS 7010B gas chromatograph quadrupole mass spectrometer/mass spectrometer (Agilent Technologies, Santa Clara, CA) and LC–MS/MS Exion HPLC 6500 Q-Trap triple-quadrupole mass spectrometer (AB Sciex, Framingham, MA).

### Standard solution preparation

High-concentration pesticide stock standard solutions were prepared from the purest analytical material commercially available, typically ≥ 95%. In general, stock standard solutions were prepared in the range of 1000–2500 μg/mL in acetone for GC–MS/MS compounds, and in either 100% acetonitrile or 100% methanol for LC–MS/MS compounds. From these, intermediate and spiking standard solutions were prepared respectively at 50 μg/mL and 1 μg/mL. Calibration standards were prepared with each sample set at concentrations of 0.8 × , 1 × , 2 × , 3 × , and 5 × the lowest calibrated level (LCL) in pesticide-free cannabis matrix extract to compensate for ion suppression/enhancement effects.

### Sample preparation-dried cannabis flowers

Cannabis inflorescence samples (5–20 g) were homogenized in a laboratory blender. Acetonitrile (20 mL) was added to 2 g ground cannabis inflorescence sample and the mixture was extracted with a Geno-Grinder at 1750 rpm for 2 min. The tube was centrifuged at 4500 rpm for 5 min. Exactly 4 mL of the extract was added to a tube containing 1 g of dispersive C18 and shaken by Geno-Grinder at 1200 rpm for 1 min. Exactly 2 mL was transferred to an ENVI-Carb/Aminopropyl SPE containing 1 cm of Na_2_SO_4_, and eluted with 25 mL of 3:1 ACN:Toluene. The sample’s solvent was exchanged to acetone, blown down to less than 1 mL using a rotary evaporator, and 20 μL of 5 μg/mL 2,4,6-tribromobiphenyl was added as an internal standard. The sample was diluted to 1 mL with acetone. Half of the extract was transferred to a vial for GC–MS/MS analysis. The remaining portion’s solvent was exchanged to acetonitrile with solvent evaporator, brought to approximately 0.1 mL. Twenty microliters of isoprocarb 5 μg/mL was added as an internal standard, which was then diluted to 0.5 mL with acetonitrile and brought to 1 mL with H_2_O. The sample was filtered using a 1-cc plastic syringe and a 0.2-µm filter and transferred to a vial for LC–MS/MS analysis.

### Instrument conditions

(a) LC–MS/MS—Sample analysis was carried out using a 6500 Q-Trap LC-MSMS (AB Sciex). Analyst version 1.6.3 (AB Sciex) and MultiQuant version 3.0.2 (AB Sciex) software were used for instrument control and data analysis, respectively. A Kinetex C18 column (2.1 × 50 mm, 2.6 μm) was used and maintained at 30 °C. The source was maintained at 550 °C. The following gas parameters were used: curtain gas, 35 psi; collision gas, 9psi; ion spray voltage, 5500 V; ion source gas 1, 50 psi; ion source gas 2, 55 psi. The injection volume was 1 μL. The mobile phases were water methanol (95 + 5) + 10 mM formic acid + 10 mM ammonium formate (A) and water–methanol (5 + 95) + 10 mM formic acid + 10 mM ammonium formate (B). The flow rate was 0.7 mL/min. The following elution gradient was used: 0–20 min, 0% B increasing to 100% B; 20–24.50 min, 100% B; 24.50–24.60 decreasing to 0% B then held from 24.60 to 25 min. Analysis was carried out by positive electrospray ionization using retention time-scheduled multiple reaction monitoring (MRM) to acquire two transitions (quantitative and qualitative) for each analyte. A partial list of these transition masses for both the LC − MS/MS and GC − MS/MS methods can be found in Table S1 and S2, respectively.

(b) GC–MS/MS—an Agilent 7010B GC–MS/MS carried out sample analysis. Mass Hunter software (Agilent) was used for instrument control and data analysis. The injection port was a multi mode injector (MMI) maintained at 250 °C. The liner was an inert double tapered splitless liner (Agilent # 5190–3983). The injection volume was 1 μL in splitless mode. Helium carrier gas was maintained at a constant flow of 1.0 mL/min. ZB-Multiresidue-1 capillary columns were used (2 columns; each of 15 m × 0.25 mm × 0.25 μm) (Phenomenex # 7EG-G016-11-CI) with backflush procedure at mid-column. The front column was fitted with a 1-m retention gap of the same stationary phase. The oven temperature was maintained at 60 °C for 1 min, ramped to 120 °C at 40 °C/min, then ramped to 310 °C at 5 °C/min with a 11.5-min hold (total run time: 52 min). The temperature of the MS source was maintained at 300 °C and the transfer line at 305 °C. Nitrogen was used as the collision gas at a flow of 1 mL/min. Analysis was carried out by electron impact ionization using dynamic MRM to acquire at least two transitions (quantitative and qualitative) for each analyte.

### Validation criteria

Quantitative validation data must show that specific pesticide/matrix combinations can be accurately quantitated at the LCL deemed fit for purpose, the lowest value for the method being 0.01 µg/g. The LCL for each pesticide was determined by an injection of a series of matrix-matched standards. The LCL was deemed acceptable if the signal of the LCL peak height to the height of the surrounding noise was at a minimum of 5:1 ratio for two transitions for the GC–MS/MS and LC–MS/MS. This ratio is the relative intensity of the quantifying ion’s response compared to the qualifying ion’s response. Ion ratios must be within permitted tolerances to be acceptable (Table 2).

**Table 2:** Permittable tolerance of quantifying ion responses compared to the qualifying ion relative intensity

Relative intensity (% of base peak)	Permitted tolerance
> 50%	± 20%
> 20 to 50%	± 25%
> 10 to 20%	± 30%
≤ 10%	± 50%

In addition, 5 replicate spikes at the LCL must meet method performance criteria of mean recoveries in the range of 70–120% with an RSD ≤ 20%. Exceptionally, a mean recovery below 70% may be acceptable if the recovery is consistent with an RSD ≤ 20% (European Commission, 2019).

The accuracy and precision of the pesticide recoveries were measured by spiking blank cannabis inflorescence matrix at the LCL (*n* = 5), 3 × LCL (*n* = 3) and 5 × LCL (*n* = 2). Linearity was established based on matrix-matched standards in the concentration range of 0.005–0.04 μg/mL for LC-MSMS, 0.010–0.080 μg/mL for GC-MSMS. The calibration curve generated from the standards must have a correlation coefficient (*R*^2^) greater or equal to 0.99.

### Quality control

After the method was validated, samples were analysed with quality control measures in place for each sample set to ensure the integrity of the results. Each set of samples included a reagent blank, a matrix blank, and a representative matrix spike at the LCL for quality control. A blank sample was spiked with 200 µL of 0.1 µg/mL of GC–MS/MS and LC–MS/MS spiking solutions. The spike was allowed to stand for a minimum of 30 min. The blanks and spike were then processed the same way as the samples. To compensate for matrix effects on pesticides in plant material all standards were made from pesticide free cannabis inflorescences matrix extracts with the addition of pesticides standards at various concentrations. Results were calculated using a six-point calibration curve (at concentrations of 0.8 × , 1 × , 2 × , 3 × , 5 × , and 10 × the LCL).

## Results

### Validation data

To meet the 2019 mandatory cannabis testing for 96 pesticide active ingredients (Mandatory cannabis testing for pesticide active ingredients requirements 2019) the new method was validated using more sensitive GC–MS/MS and LC–MS/MS instruments with better selectivity to detect 327 pesticides. Although most pesticides met the validation requirements for a LCL of 0.010 µg/g, 31% of pesticides (101 out of 327) did not meet the 0.010 µg/g LCL target and were validated at higher levels. These higher adjusted LCLs range from 0.02 µg/g to 0.4 µg/g (Table S3). Overall, 285 pesticides tested meet validation criteria and can confidently give a quantitative result (Table S3). While the remaining 42 pesticides did not pass the stringent quantification validation, they still met the criteria for monitoring their presence in cannabis inflorescence. When qualitatively identified in a sample, the mention ‘monitored’ is added for these 42 pesticides.

Mean recoveries in the range of 70 − 120% with a relative standard deviation (RSD) ≤ 20% between the 10 spiked replicates were achieved for over 68% of the pesticides validated (Table S3). Mean recoveries below 70% were still accepted (in the range of 30 to 69% only, lower than 30% is considered not recovered) if RSD ≤ 20% for compound recoveries at that level. Of the 285 pesticides that passed the validation, 22% adhere to this exception for lower 30–69% recoveries with an RSD ≤ 20% (Table S3). Overall, our method demonstrated good linearity for 83% pesticides attempted as the calibration curves had a correlation coefficient greater than 0.99.

It is important to note that piperonyl butoxide did not meet the validation criteria due to a large interference present in the reference material. The samples found positive were quantitated with a more targeted method with enough resolution to provide separation of the piperonyl butoxide and interfering signals to gain a better performance for this compound. A summary of the 12 additional recoveries outside of the validation (Table S4) shows piperonyl butoxide has a better average recovery of 40% at 0.01 ppm with an RSD of 21% at the low level (*n* = 6). At a higher spike concentration of 0.25 ppm an average recovery of 73% was observed with an RSD of 23% (*n* = 6). The correlation coefficient value for the curve used to calibrate these recoveries was acceptable (*R*^2^ = 0.9984). While the RSDs for these recoveries exceed the validation criteria, they provide more confidence in the ability of this method to qualitatively monitor piperonyl butoxide with an estimated concentration.

### Application of method to real-world samples

This newly expanded method was applied to real-world cannabis inflorescence samples available to Canadians across the country and used to determine if unregistered pesticide use is still prevalent in the licensed market and illicit cannabis. In total, 36 licensed samples and 24 illicit cannabis samples (Table 1) were analysed against the method’s 327 pesticides. Of the 36 licensed samples analyses, only 2 pesticide residues were quantified (Table 3), representing a 6% positivity rate, with the measured concentration at our method LCL of 0.01 μg/g.

**Table 3:** Pesticide quantified in licensed and illicit samples obtained across Canada

Source	Samples analyzed	Sample positive rate (%)	Pesticides detected	Sample detection frequency	Pesticide concentration range (µg/g)
Licensed	36	6	Dichlobenil	1	0.01
			Myclobutanil	1	0.01
**Illicit**	24	92	Abamectin	2	0.06 to 0.6
			Azoxystrobin	1	0.2
			Bifenazate	4	0.009 to 0.1
			Boscalid	1	0.04
			Carbaryl	2	0.02 to 0.06
			Chlorphenapyr	2	0.5 to 5
			Chlorpyrifos	4	0.01 to 30
			Dichlorvos	2	0.05 to 1
			Fluopyram	1	0.03
			Imidacloprid	3	0.1 to 60
			Malaoxon	1	0.009
			Malathion	1	0.2
			Myclobutanil	17	0.02 to 70
			Paclobutrazol	10	0.009 to 1
			Permethrin	3	0.1 to 0.7
			Piperonyl Butoxide	10	0.01 to 2^a^
			Pyrethrins	8	0.03 to 1
			Pyridaben	1	0.03
			Spinosad	1	0.2^a^
			Spirodiclofen	1	0.3
			Spiromesifen	2	0.2 to 1
			Spirotetramat	1	0.1^a^
			Tetramethrin	1	0.8

^a^Monitored

Pesticides were detected in 92% of Canadian illicit cannabis inflorescence samples with 23 unique pesticide active ingredients quantified (Table 3). Four pesticides and synergists: myclobutanil, paclobutrazol, piperonyl butoxide, and pyrethrins, were detected at a high sample frequency rate, 8 to 17 times in a total 24 illicit samples. One illicit sample alone contained nine different pesticide active ingredients. Illicit cannabis contained on average 3.7 different pesticides per sample, and 87% of positive samples contained more than one different pesticide. The pesticide concentrations quantified varied greatly, with chlorpyrifos, imidacloprid, and myclobutanil measured at 30, 60, and 70 μg/g, over three orders of magnitude higher that the method’s LCLs of 0.01 μg/g.

## Discussion

The main objective of this study was to streamline and expand our existing cannabis inflorescence method (Moulins et al. 2018) which was possible with more powerful instruments and enabled the addition of a GC–MS/MS quantification split. The existing modified quick, easy, cheap, effective, rugged, and safe (QuEChERS) extraction (Moulins et al. 2018) was adapted by eliminating the addition of water, salting-out, and enhanced matrix removal (EMR) clean-up steps, while dispersive C-18 replaced the C-18 column SPE for a four-fold time efficiency gain.

The method validation of the streamlined extraction and new instruments shows that all 285 pesticides meet the validation criteria based on the LCL, accuracy, precision, and linearity using matrix-matched standards. The remaining 42 pesticides that did not pass the stringent quantification validation still met the criteria for monitoring their presence in cannabis inflorescence. Cannabis inflorescence is a challenging matrix with its complex composition of oils, resins, terpenes, and cannabinoids. The power and sensitivity of modern triple quadrupole mass spectrometry enable to reach most 0.010 µg/g regulatory limits of quantification even while quantitatively detecting hundreds of pesticide and metabolite residues simultaneously. This validation data demonstrates that comprehensive testing of pesticides in cannabis inflorescence is achievable beyond the current 2019 mandatory cannabis testing for 96 pesticide active ingredient requirements (Mandatory cannabis testing for pesticide active ingredients requirements 2019) enabling the provision of essential data for future policy and regulatory decision-making. These results are in line with recent studies that successfully expanded their cannabis inflorescence pesticide method to several dozens (Dalmia et al. 2021; Daniel et al. 2019) and even hundreds (Maguire et al. 2019; Wittayanan and Chaimongkol 2021) of different pesticide residues analyzed simultaneously.

Application of this expanded method to licensed cannabis inflorescence found a 6% sample positivity rate with measured concentrations at the method’s LCL of 0.01 μg/g. Although quantified in one licenced sample, dichlobenil is not part of the mandatory cannabis testing for pesticide active ingredients list (Mandatory cannabis testing for pesticide active ingredients requirements 2019), indicating the importance of expanded multiresidue methods to generate valuable data for informed decisions regarding regulatory policies aiming Canadian cannabis users making informed choices. Despite a 6% positivity rate, the licensed Canadian cannabis sector has greatly improved with regards to presence of pesticides since the 2019 mandatory cannabis testing for pesticide active ingredients requirements, given the sample positivity rate of 30% prior to 2019 (Moulins et al. 2018).

In a striking contrast, Canadian illicit cannabis inflorescence samples show a 92% positivity rate with 23 unique pesticide active ingredients quantified and at concentrations up to three orders of magnitude higher that the method’s LCLs of 0.01 μg/g. High illicit cannabis pesticide positivity rates were also observed in other jurisdictions (Daniel et al. 2019; Cuypers et al. 2017; Schneider et al. 2014; Stempfer et al. 2021). To the authors’ knowledge, this study is the only extensive pesticide multiresidue analysis that compares pesticides in the licenced and illicit cannabis markets in a nation-wide jurisdiction where cannabis has been legalised. Albeit being a small study, our results do support the Government of Canada messaging where ‘Consuming illegal products could lead to adverse effects and other serious harms. Testing of illegal cannabis has found contaminants like pesticides and unacceptable levels of bacteria, lead and arsenic.’ (Buying Cannabis–What You Need To Know 2022).

## Conclusion

This study demonstrates a new streamlined and expanded method for the detection of 327 pesticides in cannabis inflorescence via gas chromatography—triple quadruple mass spectroscopy and liquid chromatography—triple quadruple mass spectroscopy. The validation of this method determined 285 unique pesticides can be quantified at levels ranging from 0.01 to 0.4 µg/g and 42 pesticides analyzed qualitatively. This method was applied to real world samples from both licensed and illicit markets revealing high presence and concentration of pesticides in illicit samples compared to samples from licenced market. With a 6% sample positivity rete, the licensed Canadian cannabis sector has greatly improved with regards to presence of pesticides since the 2019 mandatory cannabis testing for pesticide active ingredients requirements. As a first, this study demonstrates the importance of extensive pesticide multiresidue methods comparing pesticides in the licenced and illicit cannabis markets to generate valuable data for informed decisions regarding regulatory policies and for Canadian cannabis users making informed choices.

### Supplementary Information

**Additional file 1.**

## Data Availability

The data is available from the corresponding author on reasonable request.

## Abbreviations

QuEChERS: Quick, easy, cheap, effective, rugged, and safe
LCL: Lowest calibrated level
GC–MS/MS: Gas chromatography–triple quadruple mass spectroscopy
LC–MS/MS: Liquid chromatography–triple quadruple mass spectroscopy
RSD: Relative standard deviation
