# Supplementary material for: High levels of pesticides found in illicit cannabis inflorescence compared to licensed samples in Canadian study using expanded 327 pesticides multiresidue method
Source: J Cannabis Res. 2023 Aug 24;5:34. doi: 10.1186/s42238-023-00200-0 (PMC10463991; doi:10.1186/s42238-023-00200-0)
Supplement: Supplementary file 1 — Additional file 1. [file 42238_2023_200_MOESM1_ESM.docx]

**Table S1**: Retention times and multiple reaction monitoring (MRM) transitions of compounds determined by LC-MS/MS ^a^

|  |  | MRM Transitions | | | |
| --- | --- | --- | --- | --- | --- |
|  |  | Quantitative | | Qualitative | |
| Compound Name ^b^ | RT, min ^c^ | Precursor Ion | Product Ion | Precursor Ion | Product Ion |
| Acephate | 0.73 | 183.9 | 143.0 | 183.9 | 125.0 |
| Acetamiprid | 4.66 | 223.0 | 126.0 | 223.0 | 98.9 |
| Aldicarb | 5.47 | 208.1 | 116.0 | 208.1 | 89.0 |
| Allethrin | 15.58 | 303.1 | 135.0 | 303.1 | 91.0 |
| Avermectin B1a | 18.10 | 890.5 | 305.1 | 890.5 | 567.3 |
| Azadirachtin | 10.02 | 738.3 | 703.2 | 738.3 | 685.2 |
| Azamethiphos | 7.29 | 324.9 | 183.0 | 324.9 | 112.0 |
| Azoxystrobin | 11.64 | 404.0 | 371.9 | 404.0 | 343.9 |
| Benalaxyl | 13.63 | 325.9 | 148.0 | 325.9 | 208.1 |
| Bendiocarb | 7.33 | 224.0 | 167.1 | 224.0 | 109.0 |
| Benthiavalicarb-isopropyl | 11.92 | 381.8 | 180.0 | 381.8 | 197.1 |
| Benzovindiflupyr | 13.70 | 398.0 | 321.9 | 398.0 | 286.0 |
| Bifenazate | 12.30 | 301.0 | 170.1 | 301.0 | 198.1 |
| Bifenthrin | 18.29 | 440.1 | 181.2 | 440.1 | 166.0 |
| Bixafen | 13.33 | 413.9 | 393.8 | 413.9 | 265.9 |
| Boscalid | 11.49 | 343.0 | 307.0 | 343.0 | 271.0 |
| Buprofezin | 15.26 | 306.0 | 201.1 | 306.0 | 116.0 |
| Carbaryl | 7.98 | 202.1 | 127.1 | 202.1 | 145.1 |
| Carbetamide | 6.48 | 237.1 | 118.1 | 237.1 | 120.0 |
| Carbofuran | 7.39 | 222.1 | 165.1 | 222.1 | 123.0 |
| Chlorantraniliprole | 10.79 | 481.8 | 283.7 | 481.8 | 450.8 |
| Clofentezine | 13.74 | 303.0 | 138.0 | 303.0 | 102.0 |
| Clothianidin | 3.75 | 250.0 | 169.0 | 250.0 | 131.9 |
| Coumaphos | 13.77 | 363.0 | 227.0 | 363.0 | 211.0 |
| Cyantraniliprole | 9.34 | 475.0 | 285.7 | 475.0 | 443.9 |
| Cyazofamid | 12.79 | 325.0 | 108.0 | 325.0 | 217.1 |
| Cyflufenamid | 14.27 | 413.0 | 294.9 | 413.0 | 241.0 |
| Dichlorvos | 6.66 | 220.9 | 109.0 | 220.9 | 79.0 |
| Dimethoate | 3.91 | 230.0 | 199.0 | 230.0 | 170.9 |
| Dimethomorph 1 | 11.58 | 388.0 | 300.9 | 388.0 | 165.0 |
| Dimethomorph 2 | 11.91 | 388.1 | 300.9 | 388.1 | 165.0 |
| Dimoxystrobin | 13.10 | 327.0 | 205.1 | 327.0 | 116.0 |
| Dinotefuran | 1.85 | 203.1 | 113.1 | 203.1 | 129.1 |
| Dithiopyr | 14.96 | 401.9 | 353.8 | 401.9 | 271.9 |
| Dodemorph | 10.48 | 282.0 | 116.0 | 282.0 | 98.0 |
| Ethaboxam | 9.48 | 320.9 | 183.1 | 320.9 | 200.0 |
| Ethion | 15.75 | 385.0 | 198.9 | 385.0 | 143.0 |
| Ethoprophos | 12.27 | 243.1 | 130.9 | 243.1 | 96.9 |
| Etofenprox | 17.95 | 394.0 | 107.0 | 394.0 | 135.1 |
| Etoxazole | 16.40 | 360.1 | 141.0 | 360.1 | 113.0 |
| Fenoxycarb | 13.15 | 302.1 | 116.1 | 302.1 | 88.0 |
| Fenpyroximate | 16.73 | 422.2 | 366.0 | 422.2 | 107.0 |
| Fipronil | 13.28 | 453.9 | 367.9 | 453.9 | 289.8 |
| Flonicamid | 2.13 | 230.0 | 203.1 | 230.0 | 174.0 |
| Fludioxonil | 11.42 | 266.0 | 229.1 | 266.0 | 158.0 |
| Fluopyram | 12.32 | 397.0 | 208.0 | 397.0 | 173.0 |
| Fluoxastrobin | 12.74 | 458.8 | 188.0 | 458.8 | 102.0 |
| Halauxifen-methyl | 11.28 | 344.9 | 284.9 | 344.9 | 250.0 |
| Hexythiazox | 15.86 | 353.0 | 168.0 | 353.0 | 228.0 |
| Imazalil | 9.57 | 297.0 | 159.0 | 297.0 | 201.0 |
| Imidacloprid | 3.94 | 256.0 | 209.0 | 256.0 | 175.1 |
| Inpyrfluxam | 12.85 | 334.0 | 238.0 | 334.0 | 258.1 |
| Ipconazole | 14.78 | 334.0 | 70.1 | 334.0 | 124.9 |
| Isoprocarb ^d^ | 8.84 | 194.1 | 95.0 | NA ^e^ | NA |
| Isopyrazam | 14.54 | 360.0 | 339.9 | 360.0 | 320.0 |
| Isoxaben | 11.92 | 333.0 | 165.0 | 333.0 | 107.0 |
| Kresoxim-methyl | 13.13 | 314.0 | 222.0 | 314.0 | 235.0 |
| Malathion | 11.80 | 331.0 | 127.0 | 331.0 | 284.9 |
| Mandestrobin | 13.43 | 313.9 | 192.1 | 313.9 | 132.1 |
| Mandipropamid | 12.06 | 412.0 | 327.9 | 412.0 | 125.0 |
| Metalaxyl | 9.82 | 280.1 | 220.2 | 280.1 | 192.1 |
| Methiocarb | 10.87 | 226.0 | 169.0 | 226.0 | 121.0 |
| Methomyl | 2.40 | 163.0 | 88.0 | 163.0 | 58.0 |
| Metobromuron | 8.46 | 259.0 | 169.9 | 259.0 | 148.1 |
| Momfluorothrin | 13.60 | 403.0 | 162.0 | 403.0 | 385.9 |
| Myclobutanil | 12.03 | 289.0 | 70.0 | 289.0 | 125.0 |
| n-Octyl bicycloheptene dicarboximide 1 | 14.24 | 276.1 | 210.1 | 276.1 | 98.0 |
| n-Octyl bicycloheptene dicarboximide 2 | 14.82 | 276.2 | 210.1 | 276.2 | 98.0 |
| Novaluron | 15.49 | 492.9 | 158.0 | 492.9 | 141.0 |
| Oxamyl | 2.39 | 237.2 | 71.9 | 237.2 | 89.9 |
| Oxathiapiprolin | 12.56 | 540.0 | 500.0 | 540.0 | 522.0 |
| Paclobutrazol | 11.58 | 294.1 | 70.0 | 294.1 | 125.0 |
| Phenothrin | 17.75 | 351.1 | 183.0 | 351.1 | 128.0 |
| Phosalone | 14.80 | 368.1 | 182.0 | 368.1 | 111.0 |
| Phosmet | 10.64 | 317.9 | 160.1 | 317.9 | 133.0 |
| Phosphamidon | 7.70 | 299.8 | 127.0 | 299.8 | 226.9 |
| Pirimicarb | 7.30 | 238.9 | 72.0 | 238.9 | 182.1 |
| Prallethrin | 14.31 | 301.0 | 133.1 | 301.0 | 105.1 |
| Propiconazole | 13.53 | 342.0 | 159.0 | 342.0 | 123.0 |
| Propoxur | 7.10 | 210.1 | 111.1 | 210.1 | 168.1 |
| Pyraclostrobin | 14.08 | 388.0 | 194.1 | 388.0 | 163.1 |
| Pyrazophos | 4.25 | 221.9 | 104.0 | 221.9 | 92.0 |
| Pyrethrin I | 16.57 | 329.1 | 161.1 | 329.1 | 143.2 |
| Pyrethrin II | 14.93 | 373.1 | 161.0 | 373.1 | 133.0 |
| Pyridaben | 17.11 | 365.1 | 147.1 | 365.1 | 132.1 |
| Pyrifenox 1 | 11.58 | 294.9 | 92.9 | 294.9 | 67.1 |
| Pyrifenox 2 | 11.98 | 294.8 | 92.9 | 294.8 | 67.1 |
| Pyriproxyfen | 15.60 | 322.0 | 95.9 | 322.0 | 185.0 |
| Quinalphos | 13.90 | 299.0 | 163.1 | 299.0 | 96.9 |
| Resmethrin | 17.60 | 339.1 | 171.0 | 339.1 | 128.0 |
| Rotenone | 13.13 | 395.1 | 213.1 | 395.1 | 192.1 |
| Sedaxane 1 | 12.15 | 332.0 | 159.0 | 332.0 | 292.0 |
| Sedaxane 2 | 12.69 | 332.1 | 159.0 | 332.1 | 292.0 |
| Spinosad 1 | 14.82 | 732.4 | 142.1 | 732.4 | 98.1 |
| Spinosad 2 | 15.37 | 746.4 | 142.2 | 746.4 | 98.1 |
| Spirodiclofen | 16.77 | 411.0 | 312.9 | 411.0 | 212.9 |
| Spiromesifen | 16.37 | 388.2 | 273.1 | 388.2 | 255.1 |
| Spirotetramat | 12.71 | 374.1 | 302.1 | 374.1 | 216.1 |
| Spiroxamine | 11.47 | 298.3 | 144.1 | 298.3 | 100.1 |
| TCMTB | 10.44 | 238.9 | 179.9 | 238.9 | 136.0 |
| Tebuconazole | 13.42 | 308.1 | 70.0 | 308.1 | 89.0 |
| Tebufenozide | 13.23 | 353.1 | 133.1 | 353.1 | 297.0 |
| Teflubenzuron | 15.41 | 380.9 | 158.0 | 380.9 | 141.0 |
| Tetrachlorvinphos | 13.07 | 366.9 | 127.0 | 366.9 | 205.9 |
| Tetramethrin 1 | 15.60 | 332.1 | 164.0 | 332.1 | 135.1 |
| Tetramethrin 2 | 15.80 | 332.2 | 164.0 | 332.2 | 135.1 |
| Tetraniliprole | 10.97 | 544.9 | 355.7 | 544.9 | 375.8 |
| Thiacloprid | 5.61 | 253.0 | 126.0 | 253.0 | 90.0 |
| Thiamethoxam | 2.80 | 292.0 | 211.1 | 292.0 | 181.0 |
| Triazophos | 13.30 | 314.0 | 162.0 | 314.0 | 119.1 |
| Trifloxystrobin | 14.81 | 409.0 | 186.1 | 409.0 | 145.1 |
| Zoxamide | 13.41 | 336.0 | 186.9 | 336.0 | 158.9 |

^a^ Representative partial list provided for Compliance and Enforcement purposes.

^b^ A compound followed by a number indicates the compound has multiple isomers that were detected. Each isomer was identifed seperatley but quantitated together. They are numbered according to their order of elution.

^c^ RT = retention time

^d^ Internal standard

^e^ Not applicable

**Table S2**: Retention times and MRM transitions of compounds determined by GC-MS/MS ^a^

|  |  | **MRM Transitions** | | | |
| --- | --- | --- | --- | --- | --- |
|  |  | **Quantitative** | | **Qualitative** | |
| **Compound Name ^b^** | **RT, min ^c^** | **Precursor Ion** | **Product Ion** | **Precursor Ion** | **Product Ion** |
| 2,4,6-tribromobiphenyl ^d^ | 21.91 | 390.0 | 151.0 | N/A ^e^ | N/A |
| Alachlor | 19.34 | 237.1 | 160.1 | 188.1 | 160.1 |
| Benfluralin | 14.84 | 292.0 | 264.0 | 292.0 | 206.0 |
| Bifenox | 29.75 | 340.9 | 309.9 | 189.1 | 126.0 |
| Butralin | 21.57 | 266.0 | 220.2 | 266.0 | 174.2 |
| Chlorfenapyr | 25.41 | 328.0 | 247.0 | 247.1 | 227.1 |
| Chlorfenvinphos | 22.49 | 266.9 | 159.0 | 266.9 | 81.0 |
| Chlorpyrifos | 20.92 | 198.9 | 171.0 | 196.9 | 169.0 |
| Chlorthal-dimethyl | 21.10 | 300.9 | 223.0 | 298.9 | 221.0 |
| Clodinafop-propargyl | 27.38 | 266.0 | 91.0 | 238.0 | 130.0 |
| Clomazone | 16.26 | 204.1 | 107.1 | 125.0 | 89.0 |
| Cyfluthrin | 33.96 | 198.9 | 170.1 | 162.9 | 127.0 |
| Cypermethrin | 34.57 | 164.9 | 91.0 | 163.0 | 127.0 |
| Deltamethrin 1 | 36.14 | 252.9 | 93.0 | 250.7 | 172.0 |
| Deltamethrin 2 | 36.54 | 252.9 | 93.0 | 250.7 | 172.0 |
| Diazinon | 17.28 | 137.1 | 84.0 | 137.1 | 54.0 |
| Dichlobenil | 8.35 | 171.0 | 136.1 | 171.0 | 100.0 |
| Endosulfan sulfate | 27.10 | 273.8 | 238.9 | 271.9 | 235.0 |
| Endosulfan-alpha | 23.43 | 194.9 | 160.0 | 194.9 | 159.0 |
| Endosulfan-beta | 25.54 | 206.9 | 172.0 | 194.9 | 124.9 |
| Etridiazole | 10.28 | 211.1 | 183.0 | 183.0 | 140.0 |
| Fenarimol | 31.35 | 219.0 | 107.1 | 139.0 | 75.0 |
| Fenitrothion | 19.79 | 98.0 | 70.1 | 98.0 | 55.1 |
| Fenson | 20.80 | 141.0 | 77.1 | 267.9 | 77.1 |
| Fenthion | 20.36 | 278.0 | 109.0 | 124.9 | 47.0 |
| Fenvalerate 1 | 36.14 | 224.9 | 119.0 | 167.0 | 125.1 |
| Fenvalerate 2 | 36.52 | 224.9 | 119.0 | 167.0 | 125.1 |
| Flamprop-isopropyl | 25.93 | 276.0 | 77.0 | 156.0 | 129.0 |
| Heptanofos | 12.30 | 124.0 | 89.0 | 124.0 | 63.0 |
| Iprodione | 28.77 | 313.8 | 55.9 | 243.9 | 187.0 |
| Metazachlor | 22.06 | 209.0 | 132.2 | 133.1 | 132.1 |
| Metribuzin | 18.75 | 198.0 | 82.0 | 198.0 | 55.0 |
| Napropamide | 23.93 | 128.0 | 100.1 | 128.0 | 72.1 |
| Oxadiazon | 24.61 | 174.9 | 112.0 | 174.9 | 76.0 |
| Oxadixyl | 24.61 | 174.9 | 112.0 | 174.9 | 76.0 |
| Parathion-methyl | 18.56 | 125.0 | 47.0 | 262.9 | 109.0 |
| Permethrin 1 | 32.60 | 183.1 | 168.1 | 165.0 | 91.0 |
| Permethrin 2 | 32.85 | 183.1 | 168.1 | 165.0 | 91.0 |
| Piperonyl butoxide | 28.17 | 176.1 | 131.1 | 176.1 | 117.1 |
| Procymidone | 22.79 | 284.8 | 96.0 | 282.8 | 96.0 |
| Propargite | 28.00 | 135.0 | 135.1 | 135.0 | 107.1 |
| Propetamphos | 16.82 | 235.8 | 166.0 | 138.0 | 64.0 |
| Propham | 9.86 | 178.9 | 93.0 | 178.9 | 137.1 |
| Quintozene | 16.68 | 248.8 | 213.8 | 141.9 | 106.9 |
| S-Kinoprene | 20.68 | 149.0 | 93.0 | 149.0 | 77.0 |
| Sulfotep | 14.50 | 201.8 | 145.9 | 237.8 | 145.9 |
| Triadimefon | 21.04 | 208.0 | 181.1 | 208.0 | 111.0 |
| Triadimenol | 22.57 | 168.0 | 70.0 | 128.0 | 65.0 |
| Vinclozolin | 19.03 | 197.9 | 145.0 | 187.0 | 124.0 |

^a^ Representative partial list provided for Compliance and Enforcement purposes.

^b^ A compound followed by a number indicates the compound has multiple isomers that were detected. Each isomer was identified seperately but quantitated together. They are numbered according to their order of elution.

^c^ RT = retention time

^d^ Internal standard

^e^ Not applicable

**Table S3**. Validation data for compounds validated in cannabis inflorescence ^a^

|  |  |  |  | LCL (*n* = 5) | | 3 x LCL (*n* = 3) | | 5 x LCL (*n* = 2) | |  |
| --- | --- | --- | --- | --- | --- | --- | --- | --- | --- | --- |
| Compounds | Class | Group | LCL, µg/g | Mean rec. % | RSD % | Mean rec. % | RSD % | Mean rec. % | RSD % | R² |
| Avermectin B1a | Acaricide (Miticide) | Abamectin | 0.1 | 58 | 23 | 54 | 9 | 41 | 13 | 0.9539 |
| Acephate | Insecticide | Organophosphate | 0.02 | 54 | 4 | 55 | 4 | 56 | 4 | 0.9990 |
| Acetamiprid | Insecticide | Neonicotinoid | 0.01 | 88 | 4 | 83 | 6 | 82 | 1 | 0.9987 |
| Alachlor | Herbicide | Chloroacetanilide | 0.01 | 91 | 25 | 85 | 6 | 85 | 4 | 0.9962 |
| Aldicarb | Insecticide | Carbamate | 0.04 | 85 | 6 | 92 | 2 | 87 | 2 | 0.9854 |
| Allethrin | Insecticide | Pyrethroid | 0.2 | 76 | 6 | 79 | 3 | 77 | 4 | 0.9936 |
| Azadirachtin | Biopesticide | Limonoid | 0.2 | 93 | 10 | 86 | 10 | 90 | 1 | 0.9830 |
| Azamethiphos | Insecticide | Organophosphate | 0.01 | 61 | 4 | 54 | 3 | 54 | 4 | 0.9991 |
| Azoxystrobin | Fungicide | Strobilurin | 0.01 | 97 | 6 | 92 | 5 | 93 | 1 | 0.9910 |
| Benalaxyl | Fungicide | Acylalanine | 0.01 | 79 | 5 | 79 | 3 | 77 | 1 | 0.9981 |
| Bendiocarb | Insecticide | Carbamate | 0.01 | 86 | 4 | 88 | 3 | 87 | 6 | 0.9965 |
| Benfluralin | Herbicide | Nitroaniline | 0.1 | 71 | 3 | 76 | 6 | 82 | 0 | 0.9904 |
| Benthiavalicarb-isopropyl | Fungicide | Carbamate | 0.01 | 78 | 4 | 73 | 3 | 71 | 5 | 0.9955 |
| Benzovindiflupyr | Fungicide | Chitin synthesis inhibitor | 0.001 | 86 | 11 | 79 | 4 | 79 | 1 | 0.9912 |
| Bifenazate | Insecticide | Hydrazine carboxylate | 0.01 | 84 | 12 | 84 | 4 | 83 | 2 | 0.9899 |
| Bifenox | Herbicide | Diphenyl ether | 0.1 | 82 | 5 | 76 | 14 | 65 | 28 | 0.9923 |
| Bifenthrin | Insecticide | Pyrethroid | 0.1 | 70 | 17 | 114 | 17 | 86 | 3 | 0.8917 |
| Bixafen | Fungicide | Pyrazolium | 0.01 | 93 | 5 | 83 | 4 | 86 | 2 | 0.9927 |
| Boscalid | Fungicide | Carboxamide | 0.01 | 87 | 11 | 83 | 2 | 84 | 1 | 0.9965 |
| Buprofezin | Insecticide | Chitin synthesis inhibitor | 0.01 | 73 | 6 | 75 | 2 | 71 | 1 | 0.9948 |
| Butralin | Herbicide | Nitroaniline | 0.01 | 73 | 6 | 79 | 6 | 84 | 1 | 0.9920 |
| Carbaryl | Insecticide | Carbamate | 0.01 | 93 | 5 | 83 | 2 | 89 | 0 | 0.9966 |
| Carbetamide | Herbicide | Carbamate | 0.01 | 90 | 5 | 82 | 4 | 81 | 4 | 0.9990 |
| Carbofuran | Insecticide | Carbamate | 0.01 | 89 | 5 | 84 | 2 | 83 | 3 | 0.9993 |
| Chlorantraniliprole | Insecticide | Amide | 0.01 | 83 | 4 | 82 | 5 | 79 | 5 | 0.9896 |
| Chlorfenapyr | Acaricide (Miticide) | Pyrrole | 0.02 | 85 | 9 | 85 | 7 | 86 | 8 | 0.9924 |
| Chlorfenvinphos | Insecticide | Organophosphate | 0.02 | 75 | 7 | 79 | 10 | 72 | 14 | 0.9954 |
| Chlorpyrifos | Insecticide | Organophosphate | 0.01 | 80 | 15 | 73 | 3 | 75 | 1 | 0.9952 |
| Chlorthal-dimethyl | Herbicide | Benzoic acid | 0.01 | 76 | 3 | 82 | 5 | 82 | 2 | 0.9905 |
| Clodinafop-propargyl | Herbicide | Aryloxyphenoxypropionate | 0.01 | 86 | 10 | 89 | 19 | 84 | 23 | 0.9928 |
| Clofentezine | Acaricide (Miticide) | Triazine | 0.01 | 80 | 5 | 76 | 2 | 73 | 1 | 0.9971 |
| Clomazone | Herbicide | Isoxazolidinone | 0.01 | 73 | 7 | 78 | 5 | 81 | 0 | 0.9901 |
| Clothianidin | Insecticide | Neonicotinoid | 0.01 | 80 | 6 | 76 | 4 | 78 | 6 | 0.9989 |
| Coumaphos | Insecticide | Organophosphate | 0.02 | 88 | 6 | 85 | 3 | 84 | 1 | 0.9975 |
| Cyantraniliprole | Insecticide | Diamide | 0.01 | 90 | 5 | 84 | 3 | 82 | 4 | 0.9994 |
| Cyazofamid | Fungicide | Imidazole | 0.04 | 89 | 4 | 88 | 7 | 85 | 3 | 0.9957 |
| Cyflufenamid | Fungicide | Anilide | 0.01 | 91 | 6 | 87 | 3 | 86 | 4 | 0.9956 |
| Cyfluthrin | Insecticide | Pyrethroid | 0.3 | 86 | 13 | 77 | 33 | 54 | 38 | 0.9730 |
| Cypermethrin | Insecticide | Pyrethroid | 0.3 | 92 | 13 | 77 | 25 | 55 | 42 | 0.9868 |
| Deltamethrin | Insecticide | Pyrethroid | 0.05 | 61 | 14 | 62 | 26 | 45 | 32 | 0.9527 |
| Diazinon | Insecticide | Organophosphate | 0.01 | 63 | 4 | 73 | 5 | 76 | 2 | 0.9966 |
| Dichlobenil | Herbicide | Benzonitrile | 0.01 | 64 | 6 | 65 | 3 | 75 | 1 | 0.9976 |
| Dichlorvos | Insecticide | Organophosphate | 0.2 | 57 | 4 | 53 | 8 | 56 | 4 | 0.9980 |
| Dimethoate | Insecticide | Organophosphate | 0.01 | 82 | 5 | 82 | 3 | 83 | 5 | 0.9990 |
| Dimethomorph | Fungicide | Morpholine | 0.01 | 74 | 4 | 70 | 2 | 69 | 5 | 0.9945 |
| Dimoxystrobin | Fungicide | Strobilurin | 0.01 | 84 | 4 | 83 | 8 | 81 | 3 | 0.9973 |
| Dinotefuran | Insecticide | Neonicotinoid | 0.01 | 70 | 4 | 69 | 1 | 74 | 4 | 0.9989 |
| Dithiopyr | Herbicide | Pyridine | 0.02 | 90 | 7 | 89 | 3 | 85 | 1 | 0.9970 |
| Dodemorph | Fungicide | Morpholine | 0.01 | 38 | 2 | 15 | 1 | 9 | 0 | 0.9798 |
| Endosulfan sulfate | Insecticide | Organochlorine | 0.02 | 79 | 6 | 83 | 17 | 71 | 20 | 0.9804 |
| Endosulfan-alpha | Insecticide | Organochlorine | 0.02 | 74 | 9 | 75 | 8 | 70 | 5 | 0.9912 |
| Endosulfan-beta | Insecticide | Organochlorine | 0.02 | 74 | 5 | 75 | 15 | 66 | 22 | 0.9926 |
| Ethaboxam | Fungicide | Thiazole | 0.01 | 57 | 6 | 56 | 3 | 53 | 3 | 0.9976 |
| Ethion | Acaricide (Miticide) | Organophosphate | 0.01 | 89 | 5 | 89 | 3 | 88 | 3 | 0.9938 |
| Ethoprophos | Insecticide | Organophosphate | 0.01 | 70 | 7 | 72 | 4 | 69 | 4 | 0.9898 |
| Etofenprox | Insecticide | Pyrethroid | 0.02 | 61 | 10 | 72 | 4 | 66 | 10 | 0.9582 |
| Etoxazole | Acaricide (Miticide) | Diphenyl oxazoline | 0.01 | 62 | 6 | 65 | 3 | 63 | 0 | 0.9885 |
| Etridiazole | Fungicide | Thiazole | 0.01 | 59 | 8 | 61 | 6 | 68 | 3 | 0.9977 |
| Fenarimol | Fungicide | Pyrimidine | 0.02 | 61 | 3 | 73 | 10 | 73 | 5 | 0.9896 |
| Fenitrothion | Insecticide | Organophosphate | 0.01 | 85 | 6 | 90 | 5 | 94 | 6 | 0.9979 |
| Fenoxycarb | Insecticide | Carbamate | 0.01 | 91 | 7 | 83 | 8 | 79 | 1 | 0.9973 |
| Fenpyroximate | Acaricide (Miticide) | Pyrazolium | 0.01 | 57 | 5 | 60 | 1 | 58 | 1 | 0.9883 |
| Fenson | Acaricide (Miticide) | Bridged diphenyl | 0.01 | 82 | 9 | 90 | 8 | 91 | 2 | 0.9927 |
| Fenthion | Insecticide | Organophosphate | 0.01 | 64 | 8 | 68 | 5 | 73 | 1 | 0.9981 |
| Fenvalerate | Insecticide | Pyrethroid | 0.02 | 66 | 7 | 74 | 18 | 64 | 26 | 0.9772 |
| Fipronil | Insecticide | Phenylpyrazole | 0.2 | 102 | 14 | 95 | 21 | 92 | 4 | 0.9808 |
| Flamprop-isopropyl | Herbicide | Aryloxyphenoxypropionate | 0.01 | 78 | 7 | 89 | 12 | 89 | 4 | 0.9924 |
| Flonicamid | Insecticide | Pyridine | 0.01 | 79 | 3 | 78 | 1 | 81 | 5 | 0.9978 |
| Fludioxonil | Fungicide | Phenylpyrrole | 0.04 | 91 | 7 | 85 | 11 | 92 | 4 | 0.9965 |
| Fluopyram | Fungicide | Pyramide | 0.01 | 91 | 6 | 86 | 3 | 85 | 2 | 0.9969 |
| Fluoxastrobin | Fungicide | Strobilurin | 0.01 | 96 | 2 | 86 | 4 | 85 | 0 | 0.9971 |
| Halauxifen-methyl | Herbicide | Picolinic acid | 0.01 | 89 | 5 | 83 | 5 | 81 | 1 | 0.9971 |
| Heptanofos | Insecticide | Organophosphate | 0.01 | 75 | 7 | 82 | 6 | 81 | 6 | 0.9974 |
| Hexythiazox | Acaricide (Miticide) | Carboxamide | 0.01 | 71 | 3 | 70 | 7 | 64 | 2 | 0.9718 |
| Imazalil | Fungicide | Imidazole | 0.01 | 40 | 3 | 31 | 1 | 30 | 1 | 0.9966 |
| Imidacloprid | Insecticide | Neonicotinoid | 0.01 | 88 | 9 | 85 | 5 | 84 | 4 | 0.9987 |
| Inpyrfluxam | Fungicide | Pyrazolecarboxamide | 0.01 | 86 | 6 | 86 | 5 | 83 | 2 | 0.9927 |
| Ipconazole | Fungicide | Triazole | 0.02 | 58 | 3 | 56 | 1 | 54 | 3 | 0.9975 |
| Iprodione | Fungicide | Dicarboximide | 0.02 | 73 | 7 | 75 | 28 | 54 | 28 | 0.9847 |
| Isopyrazam | Fungicide | Fungicide | 0.01 | 80 | 6 | 80 | 1 | 78 | 1 | 0.9974 |
| Isoxaben | Herbicide | Benzamide | 0.01 | 90 | 6 | 85 | 4 | 80 | 3 | 0.9950 |
| Kresoxim-methyl | Fungicide | Strobilurin | 0.02 | 97 | 19 | 80 | 4 | 83 | 1 | 0.9905 |
| Malathion | Insecticide | Organophosphorus | 0.01 | 94 | 7 | 90 | 5 | 86 | 4 | 0.9978 |
| Mandestrobin | Fungicide | Strobilurin | 0.01 | 78 | 6 | 78 | 3 | 79 | 1 | 0.9977 |
| Mandipropamid | Fungicide | Amide | 0.01 | 93 | 6 | 90 | 3 | 86 | 2 | 0.9971 |
| Metalaxyl | Fungicide | Acylalanine | 0.01 | 85 | 7 | 84 | 6 | 82 | 4 | 0.9988 |
| Metazachlor | Herbicide | Chloroacetanilide | 0.01 | 88 | 5 | 84 | 12 | 84 | 9 | 0.9959 |
| Methiocarb | Insecticide | Carbamate | 0.01 | 86 | 8 | 84 | 5 | 84 | 6 | 0.9983 |
| Methomyl | Insecticide | Carbamate | 0.01 | 86 | 7 | 83 | 4 | 81 | 1 | 0.9976 |
| Metobromuron | Herbicide | Urea | 0.01 | 99 | 26 | 90 | 13 | 83 | 4 | 0.9987 |
| Metribuzin | Herbicide | Triazinone | 0.02 | 79 | 8 | 76 | 6 | 77 | 4 | 0.9970 |
| Momfluorothrin | Insecticide | Pyrethroid | 0.01 | 92 | 4 | 90 | 2 | 82 | 2 | 0.9956 |
| Myclobutanil | Fungicide | Triazole | 0.01 | 82 | 8 | 77 | 6 | 75 | 5 | 0.9985 |
| Napropamide | Herbicide | Amide | 0.01 | 69 | 3 | 71 | 8 | 70 | 8 | 0.9929 |
| n-Octyl bicycloheptene dicarboximide | Insecticide | Dicarboximide | 0.01 | 74 | 9 | 76 | 2 | 74 | 2 | 0.9960 |
| Novaluron | Insecticide | Benzoylurea | 0.01 | 93 | 6 | 92 | 4 | 84 | 2 | 0.9966 |
| Oxadiazon | Herbicide | Oxydiazole | 0.01 | 72 | 5 | 79 | 6 | 80 | 3 | 0.9957 |
| Oxadixyl | Fungicide | Phenylamide | 0.01 | 88 | 8 | 85 | 7 | 89 | 6 | 0.9947 |
| Oxamyl | Insecticide | Carbamate | 0.02 | 89 | 6 | 81 | 6 | 82 | 4 | 0.9880 |
| Oxathiapiprolin | Fungicide | Oxazole | 0.01 | 91 | 5 | 86 | 2 | 81 | 4 | 0.9963 |
| Paclobutrazol | Plant growth regulator | Triazole | 0.01 | 75 | 5 | 69 | 2 | 69 | 1 | 0.9975 |
| Parathion-methyl | Insecticide | Organophosphate | 0.01 | 80 | 7 | 86 | 7 | 86 | 3 | 0.9965 |
| Permethrin | Insecticide | Pyrethroid | 0.3 | 61 | 3 | 78 | 12 | 82 | 7 | 0.9791 |
| Phenothrin | Insecticide | Pyrethroid | 0.1 | 73 | 9 | 80 | 3 | 73 | 6 | 0.9682 |
| Phosalone | Insecticide | Organophosphate | 0.01 | 90 | 4 | 87 | 4 | 83 | 1 | 0.9938 |
| Phosmet | Insecticide | Organophosphate | 0.01 | 87 | 5 | 89 | 6 | 81 | 4 | 0.9848 |
| Phosphamidon | Insecticide | Organophosphate | 0.01 | 84 | 3 | 78 | 2 | 78 | 2 | 0.9994 |
| Piperonyl butoxide | Synergist | Unclassified | 0.2 | 184 | 133 | 226 | 126 | 248 | 107 | 0.7867 |
| Pirimicarb | Insecticide | Carbamate | 0.01 | 82 | 6 | 74 | 2 | 76 | 1 | 0.9986 |
| Prallethrin | Insecticide | Pyrethroid | 0.2 | 76 | 13 | 86 | 14 | 86 | 1 | 0.9890 |
| Procymidone | Fungicide | Dicarboximide | 0.01 | 87 | 7 | 85 | 7 | 85 | 4 | 0.9914 |
| Propargite | Acaricide (Miticide) | Sulfite ester | 0.05 | 59 | 7 | 69 | 15 | 62 | 22 | 0.9914 |
| Propetamphos | Insecticide | Organophosphate | 0.02 | 86 | 7 | 88 | 6 | 91 | 0 | 0.9929 |
| Propham | Herbicide | Carbamate | 0.01 | 88 | 5 | 82 | 3 | 88 | 0 | 0.9977 |
| Propiconazole | Fungicide | Triazole | 0.02 | 57 | 2 | 56 | 3 | 55 | 4 | 0.9968 |
| Propoxur | Insecticide | Carbamate | 0.01 | 89 | 7 | 91 | 5 | 88 | 6 | 0.9958 |
| Pyraclostrobin | Fungicide | Strobilurin | 0.01 | 87 | 8 | 83 | 4 | 82 | 0 | 0.9971 |
| Pyrazophos | Fungicide | Organophosphate | 0.01 | 82 | 6 | 80 | 2 | 78 | 2 | 0.9970 |
| Pyrethrins | Insecticide | Pyrethroid | 0.1 | 74 | 11 | 77 | 3 | 76 | 4 | 0.9877 |
| Pyridaben | Insecticide | Pyridazinone | 0.01 | 69 | 6 | 74 | 4 | 64 | 6 | 0.9820 |
| Pyrifenox | Fungicide | Pyridine | 0.04 | 51 | 3 | 47 | 2 | 48 | 2 | 0.9964 |
| Pyriproxyfen | Insecticide | Juvenile hormone mimic | 0.01 | 77 | 5 | 79 | 2 | 75 | 3 | 0.9950 |
| Quinalphos | Insecticide | Organophosphate | 0.04 | 71 | 5 | 77 | 0 | 74 | 2 | 0.9944 |
| Quintozene | Fungicide | Aromatic | 0.01 | 59 | 5 | 64 | 3 | 69 | 1 | 0.9875 |
| Resmethrin | Insecticide | Pyrethroid | 0.04 | 58 | 5 | 61 | 2 | 55 | 4 | 0.9834 |
| Rotenone | Insecticide | Botanical | 0.02 | 90 | 8 | 84 | 3 | 79 | 1 | 0.9968 |
| Sedaxane | Fungicide | Pyrazole | 0.01 | 90 | 4 | 87 | 1 | 85 | 3 | 0.9965 |
| S-Kinoprene | Insecticide | Juvenile hormone mimic | 0.1 | 50 | 5 | 57 | 5 | 57 | 0 | 0.9970 |
| Spinosad [Total] | Biopesticide | Micro-organism derived | 0.01 | 0 | 1 | 2 | 2 | 2 | 1 | 0.8981 |
| Spirodiclofen | Insecticide | Tetronic acid | 0.2 | 63 | 3 | 68 | 4 | 69 | 10 | 0.9771 |
| Spiromesifen | Insecticide | Tetronic acid | 0.4 | 64 | 8 | 63 | 7 | 72 | 4 | 0.9773 |
| Spirotetramat | Insecticide | Tetramic acid | 0.01 | 37 | 3 | 31 | 3 | 33 | 6 | 0.9980 |
| Spiroxamine | Fungicide | Morpholine | 0.01 | 11 | 7 | 21 | 4 | 20 | 4 | 0.9331 |
| Sulfotep | Insecticide | Organophosphate | 0.01 | 131 | 20 | 101 | 10 | 93 | 4 | 0.9900 |
| TCMTB | Fungicide | Mercaptobenzothiazole | 0.02 | 83 | 6 | 83 | 3 | 83 | 4 | 0.9975 |
| Tebuconazole | Insecticide | Triazole | 0.02 | 61 | 4 | 58 | 3 | 58 | 2 | 0.9981 |
| Tebufenozide | Insecticide | Diacylhydrazine | 0.02 | 122 | 12 | 86 | 13 | 83 | 1 | 0.9834 |
| Teflubenzuron | Insecticide | Benzoylurea | 0.02 | 79 | 7 | 81 | 2 | 77 | 3 | 0.9962 |
| Tetrachlorvinphos | Insecticide | Organophosphate | 0.01 | 76 | 11 | 66 | 8 | 60 | 7 | 0.9972 |
| Tetramethrin | Insecticide | Pyrethroid | 0.1 | 77 | 7 | 85 | 2 | 74 | 0 | 0.9857 |
| Tetraniliprole | Insecticide | Diamide | 0.01 | 93 | 5 | 91 | 7 | 91 | 0 | 0.9957 |
| Thiacloprid | Insecticide | Neonicotinoid | 0.01 | 90 | 3 | 84 | 2 | 84 | 4 | 0.9996 |
| Thiamethoxam | Insecticide | Neonicotinoid | 0.01 | 83 | 6 | 79 | 3 | 82 | 5 | 0.9993 |
| Triadimefon | Fungicide | Triazole | 0.01 | 71 | 6 | 78 | 4 | 78 | 4 | 0.9955 |
| Triadimenol | Fungicide | Triazole | 0.02 | 67 | 7 | 70 | 8 | 70 | 6 | 0.9981 |
| Triazophos | Insecticide | Organophosphate | 0.01 | 86 | 8 | 86 | 2 | 88 | 2 | 0.9982 |
| Trifloxystrobin | Fungicide | Strobilurin | 0.01 | 90 | 5 | 89 | 1 | 89 | 2 | 0.9946 |
| Vinclozolin | Fungicide | Oxazole | 0.01 | 87 | 5 | 87 | 6 | 86 | 0 | 0.9931 |
| Zoxamide | Fungicide | Benzamide | 0.01 | 85 | 5 | 80 | 4 | 80 | 4 | 0.9934 |

^a^ Only partial representative list provided for Compliance and Enforcement purposes.

LCL = lowest calibration level

Mean rec. - mean recovery

**Table S4**: Piperonyl butoxide performance in the actual cannabis samples outside the original method validation

|  | Recoveries | |  |
| --- | --- | --- | --- |
| Spike Sample (#) | Low at 0.01ppm (%) | High at 0.25ppm (%) | R² |
| 1 | 61 | 70 | 0.9984 |
| 2 | 31 | 118 |  |
| 3 | 9.8 | 59 |  |
| 4 | 25 | 57 |  |
| 5 | 56 | 63 |  |
| 6 | 56 | 71 |  |
| **Average** | **40** | **73** |  |
| **RSD** | **21** | **23** |  |
